# Identification of NUV-244 as a PNPLA3 I148M degrading small molecule

**DOI:** 10.1016/j.isci.2025.112384

**Published:** 2025-04-08

**Authors:** Patrick Steigemann, Nico Braeuer, Vera Puetter, Nina Zablowsky, Katrin Juenemann, Franz von Nussbaum, Ralf Lesche, Nicole Dittmar, David Schaller, Zuzanna Makowska, Filippos Klironomos, Susanne Schwarz, Daniela Launhardt, Benjamin Bader, Martin Lange, Holger Steuber, Mary Helen Black, Jonathan S. Packer, Stefano Romeo, Stephan Fasler, Lisa Bedford, Frederick E. Dewey

**Affiliations:** 1 Nuvisan ICB GmbH, Müllerstrasse 178, 13353 Berlin, Germany; 2 University of Gothenburg, Gothenburg, Sweden; 3 Foresite Labs, San Francisco, CA, USA

**Keywords:** Biological sciences, Biochemistry, Cell biology, Functional aspects of cell biology

## Abstract

The PNPLA3 I148M variant is a key genetic determinant of metabolic dysfunction-associated steatotic liver disease (MASLD) and related conditions, contributing to lipid metabolism dysregulation and disease progression. To identify small molecules that modulate PNPLA3 I148M, we conducted a high-content screen of over 820,000 compounds and identified NUV-244, a potent degrader of PNPLA3 I148M in liver-derived cells. NUV-244 reduces PNPLA3 I148M levels on lipid droplets via the ubiquitin-proteasome system, involving the E3 ligase BFAR, without affecting PNPLA2. It restores lipid droplet morphology and improves cellular fitness in PNPLA3 I148M-expressing cells. These findings provide a tool to investigate PNPLA3 I148M function and offer a potential strategy for developing targeted therapies for MASLD and related diseases. By enabling selective degradation of PNPLA3 I148M, this approach expands therapeutic possibilities beyond genetic manipulation, addressing a critical need in metabolic liver disease research.

## Highlights

• NUV-244 induces PNPLA3 I148M degradation via the ubiquitin-proteasome system
• NUV-244 facilitates PNPLA3 I148M degradation involving the E3 ligase BFAR
• NUV-244 selectively reduces PNPLA3 I148M without affecting PNPLA2
• NUV-244 restores cellular fitness in PNPLA3 I148M-expressing liver cells

## Graphical abstract

**Figure:**
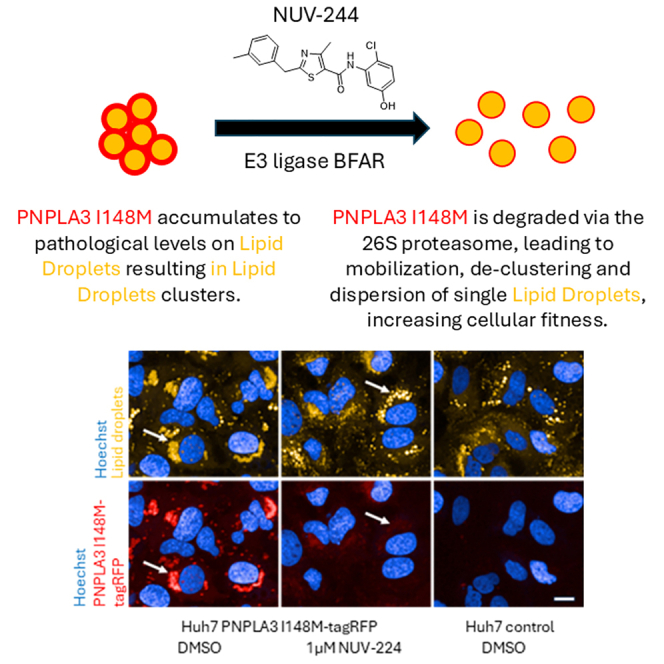

## Introduction

The prevalence of metabolic diseases, including MASLD and MASH, has been rising globally posing significant public health challenges. These conditions, which often precede more severe forms of liver damage such as fibrosis, cirrhosis, and hepatocellular carcinoma, are intimately linked to the epidemic of obesity and type 2 diabetes mellitus. Among the genetic factors contributing to the susceptibility and progression of these diseases, the patatin-like phospholipase domain-containing protein 3 (PNPLA3) I148M variant has emerged as a key determinant. The I148M variant, resulting from a single nucleotide polymorphism that substitutes methionine for isoleucine at position 148, has been robustly associated with an increased risk of MASLD, MASH, and related progressive disease, namely to cirrhosis and hepatocellular carcinoma.^1^^,^^2^^,^^3^^,^^4^^,^^5^

Lipid droplets, the cellular organelles responsible for storing neutral lipids, are central to the regulation of triglyceride homeostasis in cells. PNPLA3 is localized on the surface of lipid droplets where it contributes to the hydrolysis of triglycerides and retinyl esters and having activity as a lysophosphatidic acid acyltransferase, although its precise enzymatic activities and substrates remain areas of active investigation.^6^^,^^7^^,^^8^^,^^9^^,^^10^^,^^11^ The I148M mutation in PNPLA3 has been shown to alter the protein’s function and stability on lipid droplets, leading to aberrant lipid metabolism^12^^,^^13^^,^^14^^,^^15^ and potentially modulation of lipophagy.^16^^,^^17^ Intrinsic PNPLA3 turnover is regulated by the ER-localized E3 ligase BFAR^18^ and the PNPLA3 I148M mutation has been shown to protect PNPLA3 from degradation, leading to its accumulation on lipid droplets,^12^^,^^13^^,^^14^ which correlates with disease severity.^19^ Interestingly, the mechanism by which PNPLA3 I148M promotes lipid accumulation is not solely due to an alteration of its enzymatic function. PNPLA3 I148M also interacts with other key proteins involved in lipid metabolism, such as CGI-58 (ABHD5), which normally activates ATGL (adipose triglyceride lipase, also known as PNPLA2), a critical enzyme in lipid droplet catabolism. By sequestering CGI-58, PNPLA3 I148M prevents it from activating ATGL, reducing ATGL-mediated lipolysis, which leads to the accumulation of triglycerides in lipid droplets. This interplay between PNPLA3 I148M and CGI-58 disrupts normal lipid homeostasis and is thought to contribute to the pathological lipid accumulation observed in individuals with the I148M variant.^20^^,^^21^

Despite the growing understanding of its contribution to metabolic liver disease pathogenesis, therapeutic options that specifically target the PNPLA3 I148M variant are absent. Current treatments for MASLD and MASH have largely been supportive, focusing on lifestyle interventions and management of risk factors. However, the recent approval of THRβ agonists offers a new therapeutic option with promising safety and efficacy profiles.^22^^,^^23^ The accumulation of PNPLA3 I148M on the surface of lipid droplets contributes to its pathogenicity.^5^^,^^12^^,^^13^^,^^14^^,^^19^^,^^20^^,^^21^ Indeed, PROTAC (proteolysis targeting chimera)-mediated degradation of a Halo-tagged PNPLA3 I148M variant showed significant effect on normalization of hepatic triglyceride levels in livers of PNPLA3 I148M expressing mice^12^ and silencing of PNPLA3 using small interfering RNA lipid nanoparticles prevented development and progression of MASH and fibrosis.^24^^,^^25^ Therefore, treatments that result in the reduction or delocalization of PNPLA3 I148M from lipid droplets are of high therapeutic interest.

Given the urgent need for novel therapeutic strategies that can halt or reverse the progression of MASLD and MASH, especially in individuals harboring the PNPLA3 I148M variant, our study aimed to identify and characterize small molecule modulators capable of targeting and degrading PNPLA3 I148M. Through the application of high-content screening, we sought to identify compounds that lead to a reduction of PNPLA3 I148M on lipid droplets, aiming at a targeted small molecule-based approach to treating PNPLA3 I148M-dependent metabolic liver diseases.

We here present the identification of a small molecule PNPLA3 I148M modulator. NUV-244 is a potent small molecule to act as degrader of PNPLA3 I148M via the ubiquitin-proteasome system and involving the E3 ligase BFAR.

## Results

The PNPLA3 I148M variant is a well-known genetic determinant in MASLD and MASH. We confirmed and extended previous findings by analyzing data from the UK Biobank. Subjects with the PNPLA3 I148M variant had a significantly higher risk of progressing to cirrhosis (Figure S1A; Table S1). Among subjects with type 2 diabetes, the PNPLA3 I148M variant increased the risk of cirrhosis progression to a greater extent (Figure S1B; Table S1). These results confirm that the PNPLA3 I148M variant significantly impacts the progression of chronic liver disease, particularly to cirrhosis, both in the general population and in individuals with type 2 diabetes. Given the critical role of PNPLA3 I148M in liver disease progression, we sought to identify small molecules that could modulate PNPLA3 I148M on lipid droplets, aiming to develop targeted therapies for this variant.

### HTS for the identification of PNPLA3 I148M modulators

**Figure 1:**
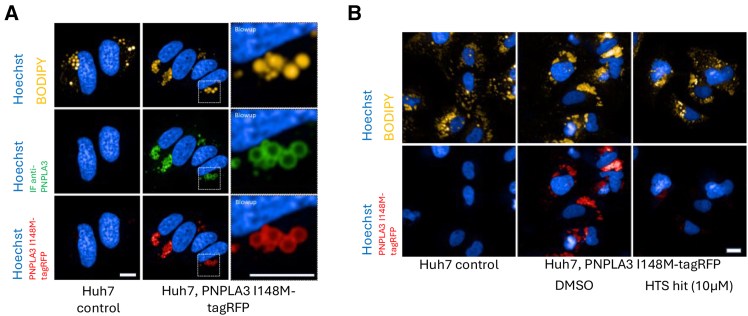
HTS for the identification of PNPLA3 I148M modulators (A) Huh7 parental control or Huh7 cells expressing PNPLA3 I148M-tagRFP were stained for PNPLA3 by immunofluorescence. In PNPLA3 I148M expressing cells, PNPLA3 I148M-tagRFP is located on the surface of lipid droplets (yellow channel) as seen by antibody staining against PNPLA3 (green channel) and the PNPLA3 I148M-tagRFP fluorescent signal (red channel). Blowup of cell overview pictures (dotted square) show PNPLA3 I148M-tagRFP being located on the surface of lipid droplets. Hoechst staining in blue. Scale bars ∼10μm. Representative images shown from *n* > 3 independent experiments. (B) Example pictures from Huh7 parental controls (left, not expressing PNPLA3 I148M-tagRFP) showing lipid droplets and no staining in the RFP channel, DMSO treated control of Huh7 cells expressing PNPLA3 I148M-tagRFP with PNPLA3 I148M-tagRFP co-localized to lipid droplets (middle) and a hit from the HTS showing strong reduction of PNPLA3 I148M-tagRFP on lipid droplets. Scale bar ∼10μm. Representative images shown from *n* > 3 independent experiments.

To establish a cellular assay for monitoring the effects of small compounds on PNPLA3 I148M localization to lipid droplets, four liver-derived cell lines were profiled. Three human hepatocellular carcinoma cell lines, Huh7, HepG2, and Hep3B, as well the human hepatic stellate cell derived cell line LX-2 were sequenced for the presence of endogenous PNPLA3 I148M mutations. Confirming literature data,^26^^,^^27^ we found Huh7, HepG2, and LX-2 cells being homozygous for PNPLA3 I148M and the Hep3B cell line being PNPLA3 WT (Figure S2A). Next, we evaluated if PNPLA3 expression can be detected in these cell lines. While purified PNPLA3 I148M protein was readily detectable using an antibody against PNPLA3 by western blot, PNPLA3 could not be detected in any of the cell lines by western blot (Figures S2B and S2D). Lipid droplet staining validated the presence of lipid droplets to different extents in all cell lines and with a significant increase upon 24h incubation in media containing 360μM oleic acid (Figure S2C), a condition that potentially increases PNPLA3 levels.^28^ However, no PNPLA3 was detectable in western blots in different cell lines fed with oleic acid using two different antibodies against PNPLA3 (Figure S2D). We conclude that PNPLA3 I148M is not expressed to a detectable protein level in different liver cell lines under the culture conditions used, which is supported by previous studies.^15^^,^^20^ Therefore, to monitor PNPLA3 I148M localization on the surface of lipid droplets, we generated a stable Huh7 cell line to express PNPLA3 I148M mutant with a C-terminal tagRFP fusion, that does not interfere with PNPLA3 protein localization.^20^ PNPLA3 I148M-tagRFP is localized to the surface of lipid droplets, as seen in cells co-stained with a lipid droplet stain and additionally validated by immunofluorescence using an antibody against PNPLA3 (Figure 1A). Additionally, PNPLA3 I148M-tagRFP shows a band with a molecular weight compatible with PNPLA3 in western blots (Figure S3A).

To establish a high-throughput compatible assay to screen for compounds that lead to reduction of PNPLA3 I148M-tagRFP on the surface of lipid droplets, Huh7 cells expressing PNPLA3 I148M-tagRFP were seeded in 1536 well microtiter plates. After 24h incubation, nuclei were detected by Hoechst staining, lipid droplets were identified by BODIPY staining and the mean intensity of PNPLA3 I148M-tagRFP on lipid droplets was quantified. A comparison to the Huh7 parental cell showed a mean signal/background (S/B) ratio of ∼12 for PNPLA3 I148M-tagRFP intensity and yielded a mean RZ’ of 0.6 (*n* = 2 plates).

HTS conditions were assessed on a ∼10K compound subset on 10 × 1536W plates. The mean RZ’ was 0.644, indicating excellent conditions for HTS (controls: Huh7 PNPLA3 I148M-tagRFP compared to Huh7 parental cell line). Using this setup, we screened a highly diverse library of >800k substances^29^ for compounds that lead to a reduction of PNPLA3 I148M-tagRFP fluorescence on lipid droplets (Figure 1B). With a mean RZ’ of 0.62 assay performance during the HTS was in the desired range. 8.711 initial hits were retested in the primary and PNPLA3 I148M-HiBiT assay (see below), and reconfirmed compounds with lead-like properties were tested for potency in the primary, HiBit, and IF assay (see below).

### NUV-244 properties and profiling

Patatin-like phospholipase domain-containing enzymes are critical regulators of triglyceride metabolism with distinct but overlapping roles in energy homeostasis. PNPLA2, also known as ATGL, is predominantly expressed in adipose tissue and plays a pivotal role in the hydrolysis of triglycerides into free fatty acids and glycerol, thereby initiating the lipolytic process. Given the close homology and shared functional domains between PNPLA2 and PNPLA3, selective targeting of PNPLA3 is paramount to avoid unintended inhibition of PNPLA2, which could disrupt normal lipid homeostasis and energy balance.

**Figure 2:**
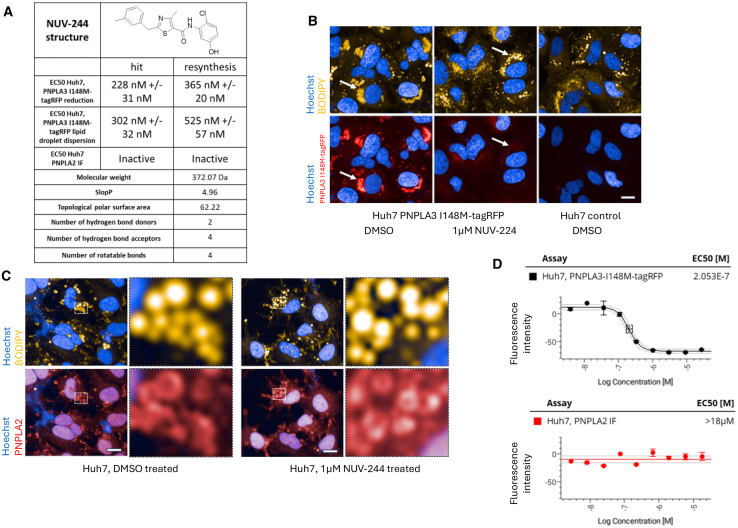
NUV-244 properties (A) NUV-244 structure and basic EC50 data. The listed properties were calculated with RDKit [RDKit: Open-source cheminformatics. https://www.rdkit.org] and satisfy Lipinski’s rule of five as well as Veber’s rule.^30^^,^^31^ (B) 1μM NUV-244 reduces PNPLA3 I148M-tagRFP from the surface of lipid droplets after 24h incubation (white arrows). Hoechst in blue, lipid droplets in yellow (upper panel). Hoechst in blue, PNPLA3 I148M-tagRFP in red (lower panel). Scale bar ∼10μm. Representative images shown from *n* > 3 independent experiments. (C) 1μM NUV-244 shows no effect on PNPLA2 staining on lipid droplets. Hoechst in blue, lipid droplets in yellow (upper panel). Hoechst in blue, PNPLA2 in red (lower panel). Blowup of cell overview pictures (dotted square) show PNPLA2 remaining on the surface of lipid droplets after NUV-244 treatment. Scale bar ∼10μm. Representative images shown from *n* = 2 independent experiments. (D) Dose-response curves for NUV-244 on Huh7 PNPLA3 I148M-tagRFP expressing cells (black curve) and immunofluorescence against PNPLA2 (red curve). Dose-response with *n* > 3 per concentration. Quantification of signal intensity on lipid droplets. Curves are normalized to DMSO treated PNPLA3 I148M-tagRFP controls (0) and parental Huh7 cells (not expressing PNPLA3, -100) or in case of PNPLA2 immunofluorescence to DMSO treated (0) and secondary antibody controls (−100). Representative data shown from *n* > 2 independent experiments.

To identify selective compounds that do not target PNPLA2 lipid droplet localization, a counter assay using immunofluorescence against endogenous PNPLA2 on lipid droplets in Huh7 cells was established and validated by PNPLA2 siRNA (Figure S4A). These efforts led to the identification of NUV-244, a thiazolecarboxamide (Figure 2A). NUV-244 was highly pure (100% as determined by LC-MS) and demonstrated dose-dependent reduction of PNPLA3 I148M-tagRFP on lipid droplets in Huh7 cells with an EC50 of 228 ± 31nM (two independent experiments) and no effect on PNPLA2 localization to lipid droplets (Figures 2A–2D). Initial computational chemistry evaluation shows that NUV-244 already has favorable physico-chemical properties (Figure 2A).

**Figure 3:**
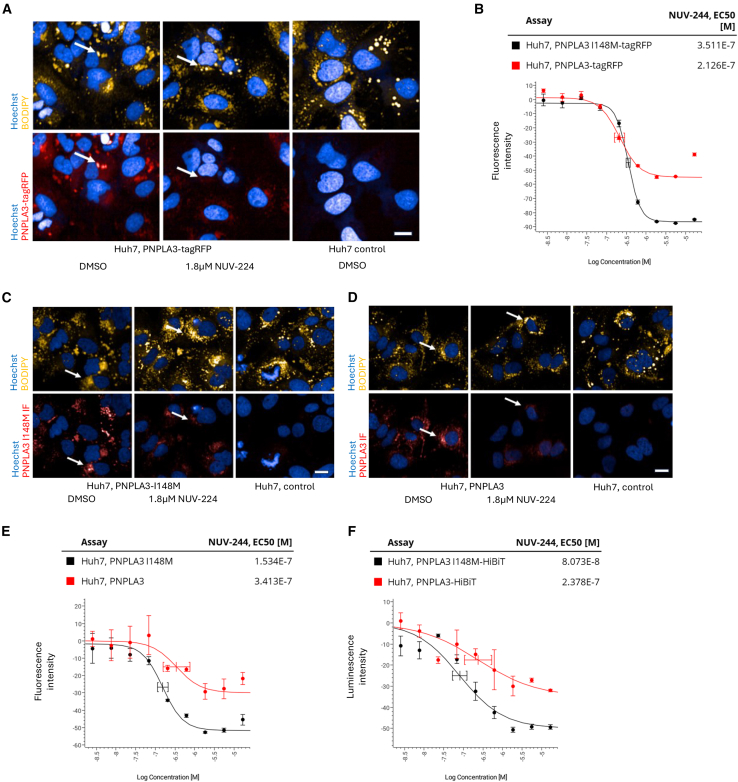
NUV-244 profiling (A) 1.8 μM NUV-244 reduces PNPLA3-tagRFP from the surface of lipid droplets after 24h incubation (white arrows). Hoechst in blue, lipid droplets in yellow (upper panel). Hoechst in blue, PNPLA3-tagRFP in red (lower panel). Scale bar ∼10μm. Representative images shown from *n* > 3 independent experiments. (B) Dose-response curves for NUV-244 on Huh7 cells either expressing PNPLA3 I148M-tagRFP (black curve) or PNPLA3-tagRFP (red curve). Dose-response with *n* > 3 per concentration. Curves are normalized to DMSO treated controls (0) and parental Huh7 cells (not expressing PNPLA3, -100). Differences in effect sizes are mainly due to higher total expression levels of PNPLA3 I148M-tagRFP as compared to PNPLA3-tagRFP. Representative data shown from *n* = 3 independent experiments. (C and D) 1.8 μM NUV-244 reduces untagged PNPLA3 I148M (c.) and PNPLA3 (d.) from the surface of lipid droplets after 24h incubation (white arrows). Hoechst in blue, lipid droplets in yellow (upper panel). Hoechst in blue, Immunofluorescence against PNPLA3 I148M (c.) or PNPLA3 (d.) in red (lower panel). Scale bar ∼10μm. Representative images shown from *n* > 3 independent experiments. (E) Dose-response curves for NUV-244 on anti-PNPLA3 immunofluorescence quantification of PNPLA3 abundance on lipid droplets in Huh7 cells either expressing PNPLA3 I148M (black curve) or PNPLA3 (red curve). Dose-response with *n* > 3 per concentration. Curves are normalized to DMSO treated PNPLA3 I148M expressing or PNPLA3 expressing controls (0) and parental Huh7 cells (not expressing PNPLA3, -100). Differences in effect sizes are mainly due to higher total expression levels of PNPLA3 I148M as compared to PNPLA3. Representative data shown from *n* = 3 independent experiments. (F) Dose-response curves for NUV-244 on Huh7 cells either expressing PNPLA3 I148M-HiBiT (black curve) or PNPLA3-HiBiT (red curve). HiBiT quantification of total protein levels. Dose-response with *n* > 3 per concentration. Curves are normalized to DMSO treated PNPLA3 I148M-HiBiT expressing or PNPLA3-HiBiT expressing controls (0) and parental Huh7 cells (not expressing PNPLA3, -100). Differences in effect sizes are mainly due to higher total expression levels of PNPLA3 I148M-HiBiT as compared to PNPLA3-HiBiT. Representative data shown from *n* = 3 independent experiments.

To confirm activity, NUV-244 was resynthesized. NUV-244 was highly pure (100% determined by LC-MS) and maintained activity in the PNPLA3 I148M-tag RFP assay (reduction of PNPLA3 I148M-tagRFP and normalization of lipid droplet morphology) and showed no activity on PNPLA2 (Figure 2A; Table 1). Next, to evaluate the specificity against PNPLA3 WT, Huh7 cells expressing PNPLA3-tagRFP were generated (Figures 3A and S3A). Like the mutant, PNPLA3-tagRFP localizes to lipid droplets in Huh7 cells and NUV-244 dose-dependently leads to a decrease of PNPLA3-tagRFP with a similar potency as for the mutant (Figure 3B). Taken together this indicates that NUV-244 does not specifically target mutant PNPLA3.

**Table 1:** Summary of NUV-244 EC50 data

Assay	NUV-244 EC50
*Huh7, PNPLA3 I148M-tagRFP*	228 nM +/− 31 nM
*Huh7, PNPLA3 I148M-tagRFP lipid droplet dispersion*	302 nM +/− 32 nM
*Huh7, PNPLA2 IF*	Inactive, >18 μM
Huh7, PNPLA3 I148M-tagRFP	365 nM +/− 20 nM
Huh7, PNPLA3 I148M-tagRFP lipid droplet dispersion	525 nM +/− 57 nM
Huh7, PNPLA2 IF	Inactive, >17 μM
Huh7, PNPLA3-tagRFP	214nM +/− 3 nM
Huh7, PNPLA3 I148M IF	124 nM +/−42 nM
Huh7, PNPLA3 IF	257nM +/− 120 nM
Huh7, PNPLA3 I148M-HiBiT	199 nM +/− 167 nM
Huh7, PNPLA3-HiBiT	302 nM +/− 90 nM
HepG2, PNPLA3 I148M-tagRFP	296 nM +/− 114 nM
Hep3B, PNPLA3 I148M-tagRFP	40 nM +/− 16 nM
LX-2, PNPLA3 I148M-tagRFP	188 nM +/− 103 nM
Huh7, PNPLA3 I148M-tagRFP, 360μM Oleic acid fed	289 nM +/− 245 nM
Differentiated OP-9 cells	2.02 μM +/− 534 nM

EC50 Data. *Italic = HTS batch data,* non-italic data from resynthesis batch, two independent determinations.

Even though C-terminal tagging is reported not to interfere with PNPLA3 localization,^20^ we established an assay to exclude the possibility that C-terminal tagging of PNPLA3 with tagRFP could alter PNPLA3 response to NUV-244 on lipid droplets. For this we generated Huh7 cell lines that express untagged variants of PNPLA3 I148M or PNPLA3 WT and visualized PNPLA3 on lipid droplets by immunofluorescence (Figures 3C, 3D, and S3A). NUV-244 effectively reduced PNPLA3 I148M and PNPLA3 from lipid droplets in a similar potency range compared to the tagRFP tagged variant (Figure 3E; Table 1). This shows that NUV-244 is also active on unmodified PNPLA3 I148M.

Next, we evaluated the effect of NUV-244 on other human hepatocellular carcinoma cells with and without endogenous PNPLA3 I148M mutations and human hepatic stellate cells. For this, we generated HepG2 (hepatocellular carcinoma, PNPLA3 I148M homozygous), Hep3B (hepatocellular carcinoma, PNPLA3 WT) and LX-2 (hepatic stellate cells, PNPLA3 I148M homozygous)^32^ cells overexpressing PNPLA3 I148M-tagRFP. In all cases, PNPLA3 I148M-tagRFP was localized to lipid droplets and we found that NUV-244 leads to dose-dependent decrease of lipid-droplet associated PNPLA3 I148M-tagRFP at similar EC50 ranges as in Huh7 cells (Table 1). This shows that NUV-244 is active in a wide range of liver-derived cells, including hepatic stellate cells. Furthermore, we tested if incubating with 360μM oleic acid, which leads to lipid droplet accumulation (Figure S2C), influenced NUV-244 activity in Huh7 cells expressing PNPLA3 I148M-tagRFP. We found similar activity in oleic acid fed cells compared to the same cells in normal medium, indicating that NUV-244 is active also in conditions in which lipid droplets are being actively remodeled (Table 1).

### NUV-244 leads to increase in lipid droplet numbers

**Figure 4:**
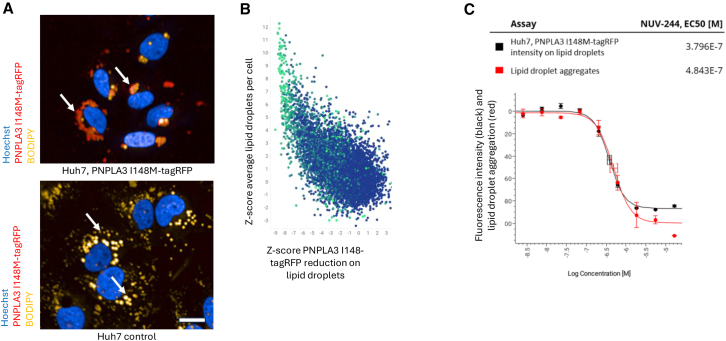
NUV-244 leads to increase in lipid droplet numbers (A) In contrast to Huh7 parental cells (lower picture), lipid droplets are clustered around the nucleus in cells expressing PNPLA3 I148M-tagRFP (white arrows in upper picture). Hoechst in blue, PNPLA3 I148M-tagRFP in red, lipid droplets in yellow. Scale bar ∼10μm. Representative images shown from *n* > 3 independent experiments. (B) HTS hits at retest (5μM concentration) stage showing scatterplot with the relationship of HTS hits leading to PNPLA3 I148M-tagRFP reduction on lipid droplets (X axis) and lipid droplet count in the same cells. Greener color (color coded for *Z* score on HiBiT level reduction: blue low/no activity, green higher activity) indicates higher activity in the Huh7 PNPLA3 I148M-HiBiT assay, showing that small molecule-induced reduction in PNPLA3 I148M levels is frequently linked to a decrease in total PNPLA3 I148M protein. (C) EC50 values of NUV-244 (resynthesis) for reduction of PNPLA3 I148M-tagRFP (black curve) on lipid droplets and the normalization of lipid droplet morphology (reduction in lipid droplet aggregates, red curve). Dose-response curves normalized to DMSO treated Huh7 (PNPLA3 I148M-tagRFP) (0) and parental Huh7 (−100) controls. Dose-response with *n* > 3 per concentration. Representative data shown from *n* = 3 independent experiments.

During the HTS we observed that PNPLA3 I148M-tagRFP expression altered the morphology of lipid droplets compared to the Huh7 parental control cells. PNPLA3 I148M-tagRFP expression leads to clustering of large lipid droplets around the nucleus and a reduction of small cytoplasmic lipid droplets, which is reflected by changes (lowering) in the number of lipid droplets scorable by the lipid droplet detection algorithm per cell (Figures 4A and 4B, Video S1). Indeed, a role for PNPLA3 I148M in the regulation of lipid droplet morphology is known.^12^^,^^13^^,^^15^^,^^16^^,^^21^^,^^33^ We speculated that reduction of PNPLA3 I148M-tagRFP on lipid droplets of Huh7 cells should therefore restore lipid droplet morphology back to the WT situation in the parental cell line. For this we analyzed all hits at the retest stage in the primary assay. Compounds that reduce PNPLA3 I148M-tagRFP on the surface of lipid droplets in Huh7 cells (Figure 4B, x-axis) lead to parallel restoration of lipid droplet numbers (Figure 4B, y axis) with a clear tendency for compounds leading to strongest PNPLA3 I148M-tagRFP reduction also leading to the strongest normalization of lipid droplet numbers in the same cells (Figure 4B). Accordingly, NUV-244 normalized lipid droplet morphology after 24h incubation with a similar EC50 as it induces reduction of PNPLA3 I148M-tagRFP on the surface of lipid droplets in the same cells, indicating that these events are interconnected (Figure 4C, Video S1). Time-lapse movies of parental Huh7 cells or Huh7 cells expressing PNPLA3 I148M treated either with DMSO control or 5μM NUV-244 for 24h before imaging showed that PNPLA3 I148M expression leads to a decrease of small and mobile lipid droplets and increases large, clustered lipid droplets, with NUV-244 being able to partially restore the appearance of small mobile lipid droplets (Video S1). This indicates that PNPLA3 I148M may have a role in the regulation of lipid droplet mobility.

Video S1. Lipid droplet in Huh7 cells over time Huh7 cells (left) and Huh7 cells expressing PNPLA3 I148M-tagRFP (right) were treated either with DMSO (upper) or 5μM NUV-244 (lower) for 24h, stained with BODIPY to visualize lipid droplets (gray) and lipid droplets were imaged for 5h with one picture taken every ∼2.5 min. PNPLA3 I148M expression leads to a reduction in small mobile lipid droplets, which are restored after 24h treatment with 5μM NUV-244. Time stamp in h:min:sec, scale bar ∼10μm.

### NUV-244 mediated degradation of PNPLA3 I148M-tagRFP occurs through the 26S proteasome and involves the E3 ligase BFAR

We observed that most hits lead to an overall decrease in PNPLA3 I148M-tagRFP intensity in cells, indicating that delocalized PNPLA3 I148M-tagRFP could become degraded. We therefore established an assay using cell lines stably expressing PNPLA3 I148M-HiBiT to evaluate the effects of the hit compounds on total cellular PNPLA3 I148M-HiBiT levels. PNPLA3 I148M-HiBiT correctly localizes to the surface of lipid droplets as revealed by immunofluorescence against PNPLA3 (Figure S5A) and shows correct band position in western blots (Figure S3A). At the retest stage of the HTS, we found that most strong HTS hits indeed also induced a reduction in the PNPLA3 I148M-HiBiT readout (Figure 4B, green colored dots). NUV-244 dose-dependently reduces the PNPLA3 I148M-HiBiT and PNPLA3-HiBiT signal at a similar potency range as it induces a reduction of PNPLA3 on lipid droplets (Figure 3F; Table 1). This effect was also seen in western blot Huh7 cells either expressing PNPLA3 I148M or PNPLA3 I148M-tagRFP (Figures S9A and S9B). This indicates that PNPLA3 reduction on lipid droplets and degradation potentially are interlinked processes.

**Figure 5:**
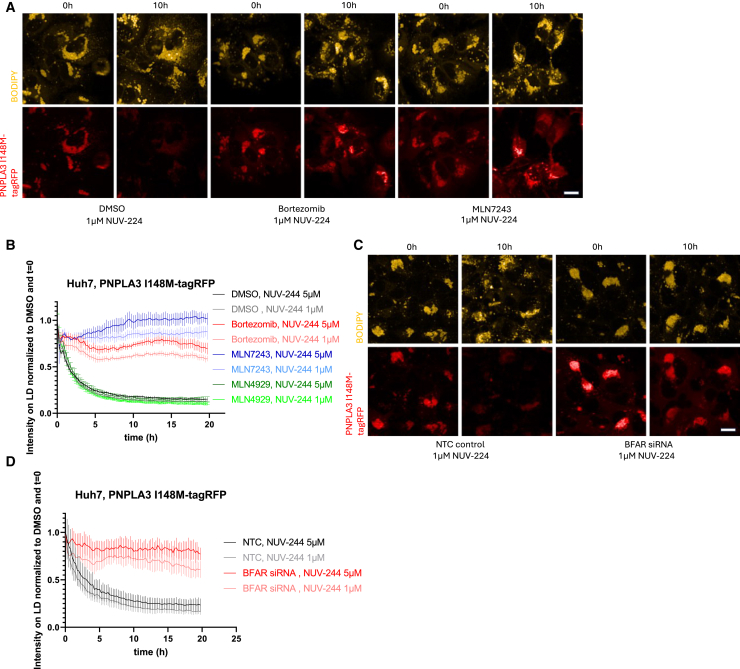
NUV-244 mediated degradation of PNPLA3 I148M-tagRFP occurs through the 26S proteasome and involves the E3 ligase BFAR (A) Huh7 cells expressing PNPLA3 I148M-tagRFP were treated with DMSO, 1μM or 5μM NUV-244 and co-treated either with DMSO or 1μM Bortezomib or 5μM MLN7243 and subjected to time-lapse imaging for 20h with one image taken every 15 min. Pictures are stills from timelapse quantified in b. at timepoint t = 0h and t = 10h. Scale bar ∼10μm. Representative images shown from *n* = 2 independent experiments. (B) Quantification of PNPLA3 I148M-tagRFP on lipid droplets shows rapid decrease in PNPLA3 I148M-tagRFP intensity on lipid droplets following treatment of 1μM or 5μM NUV-244 in cells (black and gray curves) and rescue of degradation after 1μM Bortezomib (red curves) or 5μM MLN7243 (blue curves) co-treatment but not with 5μM MLN4929 (green curves). All values are background corrected tagRFP intensity measurements on lipid droplets normalized to DMSO control and t = 0. *n* = 9 per condition. Mean and standard deviation (error bars); two independent experiments with comparable results. (C) Huh7 cells expressing PNPLA3 I148M-tagRFP were treated with non-targeting control (left) or BFAR siRNA (right) for 48h, treated with DMSO, 1μM or 5μM NUV-244 and subjected to time-lapse imaging for 20h with one image taken every 15 min. Pictures are stills from 1μM NUV-244 treated cells from timelapse quantified in d. at timepoint t = 0h and t = 10h. Scale bar ∼10μm. Representative images shown from *n* = 2 independent experiments. (D) Quantification of PNPLA3 I148M-tagRFP on lipid droplets shows rapid decrease in PNPLA3 I148M-tagRFP intensity on lipid droplets following treatment of 1μM or 5μM NUV-244 in cells treated with non-targeting control siRNA (black and gray curves) and a rescue of degradation after BFAR knockdown (red curves). tagRFP intensity measurements on lipid droplets normalized to DMSO control and t = 0. *n* = 9 per condition. Mean and standard deviation (error bars); two independent experiments with comparable results.

To evaluate the kinetics of NUV-244 mediated reduction and degradation of PNPLA3 I148M-tagRFP, we performed time-lapse studies to follow PNPLA3 I148M-tagRFP over time (Figures 5A and 5B). 1μM or 5μM NUV-244 leads to rapid onset of reduction of PNPLA3 I148M-tagRFP on lipid droplets with half maximum effect reached after approximately 2h. To evaluate if PNPLA3 I148M-tagRFP degradation is 26S proteasome dependent, 1μM Bortezomib was co-incubated with NUV-244. Proteasome inhibition prevents PNPLA3 I148M-tagRFP reduction from lipid droplets (Figures 5A and 5B). Similar data were obtained using the E1 inhibitor TAK-243/MLN7243 (5μM, Figures 5A and 5B) but not with the Nedd8 inhibitor MLN4929 (5μM, Figure 5B.), indicating that proteasomal degradation of PNPLA3 I148M-tagRFP is E1-dependent but not mediated by Culling-RING E3 ligases.^18^ A recent paper identified the E3 ligase responsible for the turnover of endogenous PNPLA3, the ER associated E3 ligase BFAR.^18^ To evaluate if NUV-244 potentially acts as a molecular glue to enhance PNPLA3 I148M polyubiquitination by BFAR, we knocked down BFAR by siRNA and found that this partially prevents PNPLA3 I148M removal from lipid droplets as well as its degradation and lipid droplet dispersion (Figures 5C and 5D, validation of siRNA knockdown in Figure S8). Taken together, these data indicate that NUV-244 acts as a molecular glue, leading to 26S-proteasome and E3 ligase BFAR dependent PNPLA3 I148M-tagRFP reduction on lipid droplets and its degradation.

To investigate the mode of action of NUV-244, we evaluated its direct binding to the PNPLA3 I148M protein using nano differential scanning fluorimetry (nanoDSF). In this assay, compounds that bind to a target protein can lead to thermal stabilization, preventing protein denaturation, which is reflected in a shift of the protein melting curve.

Our results showed that NUV-244 significantly stabilized both PNPLA3 WT and PNPLA3 I148M proteins, with ΔTm values of 7.02°C and 5.82°C, respectively. These shifts correspond to Z-scores greater than 6, indicating robust stabilization. In contrast, NUV-244 did not affect the melting curve of the PNPLA2 protein in nanoDSF assays, suggesting specific binding to PNPLA3 (Figure S6).

### NUV-244 does not affect proliferative fitness in liver cells and restores proliferative fitness in PNPLA3 I148M expressing cells

**Figure 6:**
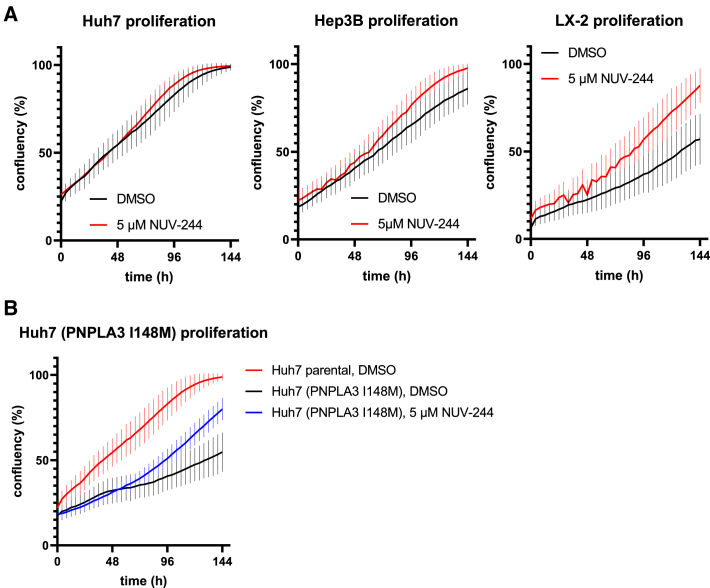
NUV-244 does not affect proliferative fitness in liver cells and restores proliferative fitness in PNPLA3 I148M expressing cells (A) Growth curves of Huh7, Hep3B and LX-2 cells were measured in Incucyte. NUV-244 at 5μM (red curves) shows no signs of toxicity compared to DMSO treated controls (black curves). Similar data was obtained with 1μM NUV-244 (data not shown). *n* = 16 per condition. Mean and standard deviation (error bars); two independent experiments with comparable results. (B) Expression of PNPLA3 I148M in Huh7 cells reduces proliferative fitness (black curve) compared to non-expressing parental control cells (red curve). Treatment with 5μM NUV-244 leads to restoration of cellular proliferative fitness (blue curve; similar data with 1μM NUV-244). *n* = 16 per condition. Mean and standard deviation (error bars); two independent experiments with comparable results. Highly significant (adjusted *p* value < 0.0001) differences in proliferation can be seen in Huh7 expressing PNPLA3 I148M treated with NUV-244 compared to DMSO control from 100h incubation onward (two-way ANOVA, not shown).

Time-lapse data show dividing cells even after prolonged NUV-244 exposition (Video S1), indicating good tolerability and limited effects on normal cellular functions. Indeed, NUV-244 has no effect on total nuclear count at effective concentrations in Huh7 cells and we frequently observed mitotic cells in the Hoechst channel even after 24h incubation (data not shown). This indicates low toxicity of NUV-244 at effective concentrations. We validated this by recording the growth curves of different cell lines upon NUV-244 incubation and found no effects of 1μM or 5μM NUV-244 on the proliferative capacity of Hep3B cells, or Huh7 and LX-2 cells compared to DMSO treated cells (Figure 6A). PNPLA3 I148M expression has been reported to sensitize cells against lipotoxicity and to induce mitochondrial dysfunction.^32^^,^^34^^,^^35^^,^^36^^,^^37^ Accordingly, we find that Huh7 cells expressing unmodified PNPLA3 I148M showed a significant reduction in proliferative fitness as compared to the parental cell line, which was restored after treatment with either 1μM or 5μM NUV-244 (Figure 6B). The time lag before NUV-244 shows effects on Huh7 (PNPLA3 I148M) cell proliferation can be explained by the time it takes for NUV-244 mediated degradation of PNPLA3 I148M to take effect (Figure 6B). Taken together, these data show that NUV-244 not only has no effect on cellular proliferation in normal liver cells but also has a positive impact on proliferative capacity in cells expressing PNPLA3 I148M, presumably by reducing a negative impact on PNPLA3 I148M expression on cellular fitness.

Finally, a recent study showed that despite showing less mRNA expression as compared to the liver, PNPLA3 I148M protein strongly accumulates in adipose tissue in humans and furthermore significantly impacts adipose tissue triglyceride composition.^38^ This indicated that targeting PNPLA3 I148M outside the liver could have beneficial effects. To test if NUV-244 acts in adipocytes, we generated mouse OP-9 cells stably expressing PNPLA3 I148M-tagRFP and differentiated these into adipocytes^39^ (DRUG-seq^40^^,^^41^ data for effective differentiation in Figures S7A and S7B, PNPLA3 I148M-tag-RFP on the surface of large lipid droplets in adipocytes in Figure S7C). NUV-244 was active in reducing PNPLA3 I148M-tagRFP in these cells, albeit with slightly less potency (Table 1).

Taken together, we here report the first identification of a small molecule compound that leads to binding, reduction, and degradation of PNPLA3 I148M in a cellular setting. Given the overall favorable profile of combining high cellular potency, acceptable physico-chemical properties and good tolerability, this compound represents an *in-vitro* tool to further study PNPLA3 I148M pathology and could also serve as a starting point for clinical development of small molecule–based PNPLA3 I148M degraders.

## Discussion

Current therapeutic strategies targeting PNPLA3 I148M primarily focus on genetic manipulations such as antisense oligonucleotides (ASOs).^22^ While these approaches are innovative and have demonstrated efficacy in reducing hepatic fat content,^12^^,^^24^^,^^42^ they come with significant limitations, including longer duration of effect induction, non-reversibility, challenges in tissue specificity and concerns about long-term safety profiles. In contrast, small molecules that directly modulate the activity of the PNPLA3 I148M protein offer distinct advantages. These include the rapid and reversible modulation of disease pathways, potential efficacy across multiple tissues beyond the liver, ease of administration, and the ability to precisely adjust therapeutic responses that can be designed to achieve higher specificity and potency. This broad tissue accessibility positions small molecule degraders as a more holistic approach to managing diseases associated with the PNPLA3 I148M variant, also potentially in combination with genetic approaches.

Moreover, small molecule approaches circumvent the uncertainties associated with genetic methods that depend on efficient cellular uptake and the intricacies of RNA interference mechanisms. Consequently, the development of small molecules capable of inducing the reduction of PNPLA3 I148M on lipid droplets and/or promoting its degradation represents a promising expansion of the therapeutic arsenal against diseases associated with the PNPLA3 I148M variant. NUV-244 is capable of delocalizing and degrading PNPLA3 I148M from lipid droplets and thereby signifies a novel mechanism of action that could potentially impact the treatment landscape for these disorders.

The data indicate that NUV-244 could act as a molecular glue to enhance the interaction between PNPLA3 I148M and the E3 ligase BFAR.^18^ This is supported by the fact that NUV-244 mediated degradation of PNPLA3 I148M is dependent on the 26S proteasome and involves the E3 ligase BFAR. While first nano-DSF data show that NUV-244 could directly binds to PNPLA3 I148M, further biochemical data are required to show that NUV-244 indeed enhances the interaction between PNPLA3 I148M and BFAR, leading to polyubiquitination and degradation of PNPLA3 I148M.

NUV-244 binding or polyubiquitination itself is not sufficient for PNPLA3 I148M-tagRFP reduction on the lipid droplet surface, as 26S proteasome inhibition, which does not interfere with the first steps of NUV-244 binding, was sufficient to prevent PNPLA3 I148M-tagFRP delocalization from lipid droplets. Therefore, a possible model could be that NUV-244 enhances PNPLA3 I148M interaction with BFAR in a molecular glue-like fashion, leading to polyubiquitination of PNPLA3 I148M on the surface of lipid droplets. Polyubiquitinated PNPLA3 I148M then could get extracted from the surface of lipid droplets to the cytoplasm and subsequently degraded by the 26S proteasome. Taking all this together, we hypothesize that NUV-244 acts in a molecular glue-like fashion to enforce PNPLA3 I148M interaction with the E3 ligase BFAR to enhance PNPLA3 I148M degradation. Further biophysical experiments and structure determination will help to elucidate on the exact function of NUV-244 as a molecular glue between BFAR and PNPLA3 I148M.

The exact molecular function of PNPLA3 is under investigation and despite the PNPLA3 I148M mutation has been identified as a strong genetic determinant for liver disease it is not completely clear which effect this mutant has on PNPLA3 and lipid biology. The phenotype of PNPLA3 I148M expression is different from PNPLA3 knockout or knockdown,^6^^,^^13^^,^^24^^,^^42^^,^^43^^,^^44^^,^^45^ indicating that the PNPLA3 I148M mutation infers a specific disease driving functional change to the protein. PNPLA3 I148M confers resistance against degradation and accumulates on lipid droplets^12^^,^^13^^,^^14^^,^^19^^,^^20^ and prevents activation of PNPLA2.^20^ Therefore, it is possible that PNPLA3 I148M exerts its adverse effects mainly by overaccumulation on lipid droplets. While it has been shown, that PNPLA3 I148M influences lipid droplet morphology, we here show that expression of PNPLA3 I148M leads to reduction in the number of small mobile lipid droplets, a phenotype that is rescued after incubation with NUV-244, indicating that accumulation of PNPLA3 I148M on lipid droplets impacts on lipid droplet mobility.

As NUV-244 enhances PNPLA3 I148M degradation by BFAR and restores cellular fitness in PNPLA3 I148M expressing cells, it is likely that NUV-244 will have positive effects in an *in vivo* MASH model setting. With NUV-244 already showing good potency in liver and hepatic stellate cells^32^ and no signs of toxicity, NUV-244 is a favorable starting point for further development into a clinical candidate. Structure-activity relationship studies will lead to the generation of an *in vivo* candidate molecule with further optimized potency, selectivity, and PK properties. As pharmacodynamic biomarker PNPLA3 I148M levels in livers could be utilized. Several *in vivo* PNPLA3 I148M driven MASH models have been reported and it will be interesting to see how NUV-244 performs in these settings.^5^^,^^12^^,^^14^^,^^42^ Alternatively, iPSC derived MASH models could be used to characterize the potential of NUV-244 to normalize PNPLA3 I148M disease driving effects.^35^^,^^46^^,^^47^ On the other hand, recent research also shows that PNPLA3 I148M induces hepatic mitochondrial dysfunction^36^ and influences lipophagy^16^ and it will be interesting to see to which extent NUV-244 can normalize these effects.

NUV-244 is a tool compound for facilitating PNPLA3 research but also represents a favorable starting point for further clinical development. One major concern for the development of PNPLA3 I148M modulators is their selectivity against the closely related patatin-like phospholipase PNPLA2, which plays a critical role in triglyceride metabolism.^48^ NUV-244 does not show binding to PNPLA2 in biophysical experiments, nor does it show delocalization or degradation of PNPLA2 in a cellular setting, indicating selectivity of NUV-244 against PNPLA2. This can be followed up by proteomics studies with NUV-244 treated cells to further elucidate on the specificity for PNPLA3 I148M degradation. In contrast, NUV-244 does not seem to be selective against the PNPLA3 WT protein, as it similarly leads to degradation of PNPLA3 WT in the tested assay systems. Missing selectivity against PNPLA3 WT is mainly of relevance in PNPLA3 I148M heterozygous patients, as in a PNPLA3 I148M homozygous background NUV-244 would act to reduce PNPLA3 I148M disease driving activity and in the case of complete degradation would resemble a PNPLA3 null situation, which shows no association with fatty liver disease under specific lipogenic conditions.^13^^,^^43^^,^^44^^,^^49^ However, a recent paper shows that PNPLA3 acts as a triglyceride lipase and loss of function potentially contributes to liver disease upon lipogenic stimulation.^49^ On the other hand, PNPLA3 I148M acts by evading degradation, accumulates on lipid droplets and prevents the activity of PNPLA2,^12^^,^^13^^,^^14^^,^^19^^,^^20^^,^^21^ so one possibility is that PNPLA3 I148M is disease driving at least partly due to its massive accumulation on lipid droplets. Therefore, a small molecule degrader that only reduces but not fully eliminates PNPLA3 I148M and PNPLA3 WT in heterozygous conditions could already have a therapeutic effect without conferring side effects through the generation of a PNPLA3 null situation.^49^ Also, NUV-244 could be dosed to obtain beneficial effects by reduction of PNPLA3 I148M while at the same time maintaining sufficient levels of PNPLA3 WT in a heterozygous situation to prevent adverse effects by too strong PNPLA3 reduction. Furthermore, structure activity studies on this or other hit compound classes could aim to generate PNPLA3 I148M selectivity against PNPLA3 WT.

Nevertheless, the main concern with targeting both the mutant and WT forms of a gene is the potential for unintended consequences on normal physiological processes. Since PNPLA3 KO mice do not exhibit significant adverse effects under normal conditions,^43^^,^^44^^,^^50^ this suggests that reducing PNPLA3 activity might not have detrimental effects on liver function. However, long-term effects and potential impacts on other tissues or metabolic processes need careful evaluation.

The discovery of NUV-244 as a targeted modulator of PNPLA3 I148M offers a promising therapeutic avenue for MASLD and MASH, diseases currently lacking specific treatments. The immediate next steps involve optimizing its pharmacological attributes, including potency, selectivity, and pharmacokinetic properties. These efforts will not only validate NUV-244’s therapeutic potential but also shed light on its pharmacokinetics and pharmacodynamics in relevant disease models.

By directly targeting the pathological mechanism at the molecular level, NUV-244 offers a promising new direction for anti-PNPLA3 I148M therapeutic development. This research paves the way for future studies aimed at harnessing the full potential of small molecule modulators in combating genetic variants implicated in metabolic disorders, with the goal of improving patient outcomes in MASLD, MASH, and beyond.

### Limitations of the study

While our findings demonstrate the efficacy of NUV-244 in degrading PNPLA3 I148M *in vitro*, the absence of *in vivo* studies limits our ability to assess the compound’s pharmacokinetics, toxicity, and therapeutic potential in a living organism. Further *in vivo* validation will be essential to confirm the translational relevance of NUV-244 as a potential therapeutic. Although we identified NUV-244 as a potential molecular glue that facilitates the degradation of PNPLA3 I148M, a more comprehensive understanding of its molecular interactions and glue-like activity is required. Further mechanistic studies are necessary to elucidate the precise binding interactions and pathways involved in its action.

## Resource availability

### Lead contact

Further information and requests for resources and reagents should be directed to and will be fulfilled by the Lead Contact, Patrick Steigemann (Patrick.Steigemann@nuvisan.com).

### Materials availability

Reagents and cell lines generated in this study are available from the lead contact upon request. This study did not generate new unique plasmids or mouse lines.

### Data and code availability

• **Data:** The datasets supporting the findings of this study are available from the lead contact upon reasonable request.
• **Code:** This study did not generate any new custom code or computational models.
• **Other:** Any additional information required to reanalyze the data reported in this paper is available from the lead contact upon request.

## Acknowledgments

The authors would like to thank Daniel Linden for helpful discussions on the manuscript.

## Author contributions

Conceptualization: P.S., S.R., L.B., and F.E.D.; methodology: P.S., N.B., and V.P.; investigation: P.S., N.B., V.P., N.Z., K.J., R.L., N.D., Z.M., F.K., S.S., M.H.B., and J.S.P.; formal analysis: P.S., N.B., V.P., N.Z., K.J., R.L., N.D., D.S., Z.M., F.K., S.S., M.H.B., and J.S.P.; writing – original draft: P.S.; writing – review and editing: P.S.; supervision: P.S., F.V.N., B.B., H.S., S.R., S.F., L.B., and F.E.D.; Resources: B.B., H.S., L.B., and F.E.D.; funding acquisition: F.V.N., L.B., and F.E.D.

## Declaration of interests

P.S., N.B., V.P., N.Z., K.J., F.V.N., R.L., N.D., D.S., Z.M., F.K., S.S., B.B., and H.S. are or were employees of Nuvisan ICB GmbH, Berlin, Germany. M.H.B., J.S.P., L.B., and F.E.D. are or were employees of Foresite Labs, USA. S.F. and S.R. were consultants for Foresite Labs, USA.

## STAR★Methods

### Key resources table

**Table:** 

REAGENT or RESOURCE	SOURCE	IDENTIFIER
**Antibodies**		

Anti-PNPLA3 C-8	Santa Cruz	SC-390252
Anti-PNPLA3	Millipore	MABS2174
Anti-HSP90	Cell Signalling	#4877
Anti-PNPLA2	Sigma Aldrich	HPA055173
Anti-BFAR	Antibodies.com	A16152
Anti-CHOP	Cell Signalling	#2895

**Experimental models: Cell lines**		

Huh7	Elabscience	
Hep3B	Elabscience	
HepG2	Elabscience	
LX-2	Elabscience	
OP-9	Elabscience	

**Oligonucleotides**		

Primers for PNPLA3: F1 (CCAACAACCCTTGGTCCTGT) and R1 (GGGTAGCCTGGAAATAGGGC)	TIB Molbiol	custom
PNPLA2 siRNA	Dharmacon	#L-009003-01-005
BFAR siRNA	Dharmacon	# L-004386-00-0005

**Recombinant DNA**		

Lentiviral constructs for PNPLA3 expression	Sirion Biotech	custom

**Software and algorithms**		

Genedata Screener	Genedata	
Graphpad prism	Graphpad	
Harmony Software	Revvity	
Prism	GraphPad	

### Experimental model and study participant details

#### Association studies

We analyzed the UK Biobank dataset Association of PNPLA3 I148M with Chronic Fatty Liver Disease Progression in the UK Biobank. This dataset comprises genotype and phenotype information on over 500,000 individuals from the general population in the United Kingdom. This research was conducted using the UK Biobank resource under application number 44424. For genotyping, imputed genotypes for the UK Biobank were retrieved from the UK Biobank data showcase (category 263, data field 22828). Genetic analyses were limited to unrelated subjects of European ancestry within the 3rd-degree of relatedness, identified using HAIL software (https://github.com/hail-is/hail). Subjects whose genetic sex (UKB data field 22001) did not match their self-reported sex (UKB data field 31) were excluded. The top 6 principal components (PCs), calculated using PCA within the 3-rd degree unrelated European-ancestry subset, were included as covariates in the regression models. Chronic fatty liver disease case-control phenotypes were defined using heuristics applied to hospital episode statistics and self-report illness codes. Cases must have had at least one of: ICD-10 B18, C22.0, K70, K72.1, K73, K74.0-K74.2, K74.6, K75.8, K76.0, K76.6-K76.7; ICD-9 155.0, 456.2, 571.0, 571.2, 571.4, 571.50, 571.51, 571.58, 571.59, 571.8, 572.3, 572.4; UKB self-report (non-cancer) 1158, 1579-1580, 1604. Controls were individuals without the aforementioned CLD diagnoses or self-report codes, and who also did not have conditions such as ICD-10 I85, K76.9; ICD-9 456.0, 456.1; UKB self-report codes (non-cancer) 1141, 1155-1157. In addition to the requirements above, cases and controls must not have had any of: ICD-10 K74.3, K75.4; ICD-9 571.6; UKB self-report (non-cancer) 1475, 1506.

Subjects were allocated into experimental groups based on their CLD status. In the overall analysis (Figure 1A), the Kaplan–Meier “number at risk” table shows 7,830 participants at baseline. In the subgroup analysis for subjects with type 2 diabetes (Figure 1B), 1,423 participants were included at baseline. Progression was analyzed from a subject’s first inpatient CLD diagnosis to a composite endpoint, which included inpatient cirrhosis (ICD-10 B18, C22.0, K70, K72.1, K73, K74.0-K74.2, K74.6, K75.8, K76.0, K76.6-K76.7; ICD-9 155.0, 456.2, 571.0, 571.2, 571.4, 571.50, 571.51, 571.58, 571.59, 571.8, 572.3, 572.4; UKB self-report codes 1158, 1579-1580, 1604) or hepatocellular carcinoma (ICD-10 C22, ICD-9 155.0), as well as procedures such as drainage of ascites (OPCS-4 T46.1, T46.2), liver transplant (OPCS-4 J01), or liver-related mortality (ICD-10 K70-K77). Progression was assessed using a Cox proportional hazards model, with the following covariates: age at CLD diagnosis, sex, genotyping chip, and ancestry PCs 1-6.

#### Cell lines and culture conditions

Huh7 (PNPLA3 I148M homozygous), HepG2 (PNPLA3 I148M homozygous), Hep3B (PNPLA3 WT), LX-2 (PNPLA3 I148M homozygous), and OP9 cells were acquired from Elabscience Biotechnology (Wuhan, China). Huh7 and Hep3B cells were cultured in RPMI1640, while HepG2 and LX-2 were cultured in DMEM (Gibco). All media were supplemented with 10% heat-inactivated fetal calf serum (Merck, FBS Superior, PAA Laboratories by GE Healthcare, Little Chalfont, UK) and 1% Penicillin/Streptomycin (Sigma-Aldrich, St. Louis, MO, USA). OP9 cells were cultured in MEM (Gibco) containing 20% FBS and 1% Penicillin/Streptomycin. Cells were maintained at 37°C, 5% CO2, and 95% air atmosphere.

Huh7, HepG2, Hep3B and LX-2 are of male origin. To confirm the genotype of PNPLA3 at amino acid position 148, the respective genomic region was amplified using Primers F1 (CCAACAACCCTTGGTCCTGT) and R1 (GGGTAGCCTGGAAATAGGGC), followed by Sanger Sequencing using the same primers. Furthermore, cells were authenticated by STR analysis (DSMZ Brunswick, Germany or Eurofins Genomics, Germany). Mycoplasm contamination was tested by MycoAlert (Lonza).

#### Lentiviral transduction

**Lentiviral Vectors** for expression of PNPLA3 WT or PNPLA3 I148M, either untagged or C-terminally tagged with HiBiT or tagRFP (Sirion Biotech, Germany) were added to cells and stable integration of constructs was achieved by culturing transduced cells in media containing 0.5 μg/mL puromycin for 48 hours post-transduction.

### Method details

#### High-throughput screening (HTS)

Huh7 cells expressing PNPLA3 I148M-tagRFP and Huh7 parental controls were seeded at 1500 cells/well in 6 μL of culture medium into compound or DMSO containing (60nl, 1mM in DMSO, 10μM final concentration) Screenstar 1536-well plates (Greiner Bio-One, Germany). Plates were incubated for 24 hours at 37°C and 5% CO2. Cells were fixed in 1% paraformaldehyde (Electron Microscopy Science, USA) and stained with 38 nM BODIPY (Invitrogen, USA) and 2 μg/mL Hoechst 33342 (Biotium, USA).

For dose-response cells were seeded at 4000 c/30 μl per well in 384 well plates (Revvity, Waltham, MA, USA, PhenoPlate 384-well microplate, #6057308) and stored in a 37°C incubator with 5% CO2 and 95% humidity overnight. Compounds, serially diluted, and DMSO controls were added, and cells were incubated for another 24h. Cells were fixed and stained with a mixture of Paraformaldehyde (final 1%, Electron Microscopy Science), BODIPY (final 38 nM, Invitrogen) and Hoechst 33342 (final 2 μg/ml, Biotium) diluted in PBS for 30 min at room temperature in the dark (no wash off).

Confocal images were acquired using the Opera Phenix system (Revvity, USA) with a 10x, 20x or 40x water objective and excitation at 405 nm (Hoechst), 488 nm (BODIPY), and 532 nm (PNPLA3 I148M-tagRFP).

Images were analyzed using Harmony (Revvity) and MetaXpress software (Molecular Devices, USA) to quantify lipid droplet morphology and PNPLA3 I148M-tagRFP intensity.

Assay plates were evaluated based on Z’ scores (>0.6) and signal-to-background ratio (>12) using Genedata Screener® for HTS and Condoseo modules (Genedata, Switzerland). Normalization, quality control, and fitting curves for EC50 determination of tested compounds were performed with Genedata Screener® for high-content screening and Genedata Condoseo modules (Genedata AG, Basel, Switzerland). More than 820K cpds were screened on 791 1536W plates in 11 runs covering three weeks of primary HTS. Automated image analysis routines were set up to quantify the number, morphology and intensity of nuclei, lipid droplets and the intensity of PNPLA3 I148M-tagRFP on lipid droplets. Toxic or autofluorescent compounds were excluded from the hitlist based on nuclear count, area, and intensity measurements as well as lipid droplet staining intensity compared to control cells.

#### HiBiT assay for PNPLA3 quantification

Cells were plated at 6000 cells/well in white 384-well low-volume plates (Corning #3826, USA) in 20 μL of culture media and treated with serial dilutions of compounds in DMSO or DMSO control for 24 hours. Cells were incubated for 24 h at 37°C in a 5% CO2 and 95% air incubator. 12μl Nano-Glo HiBiT reagent (Nano-Glo HiBiT lytic detection system, Promega, Fitchburg, WI, USA) was then added for 10 minutes at room temperature in the dark to lyse cells. Subsequently, luminescence intensity was measured in a ViewLux™ plate reader (Revvity, Waltham, MA, USA). Normalization, quality control, and fitting curves for EC50 determination of tested compounds were performed with Genedata Screener® for high-content screening and Genedata Condoseo modules (Genedata AG, Basel, Switzerland).

#### PNPLA2 siRNA knockdown and validation

For siRNA knockdown, cells were seeded at 4000 c/40 μl per well in a 384 well plate (Revvity, Waltham, MA, USA, PhenoPlate 384-well microplate, #6057308) and incubated with 10 nM siRNA and Lipofectamine RNAiMAX (Thermo Fisher, Waltham, MA, USA, 1:1000) or control (lipid only, non-targeting-control siRNA, PLK1 siRNA) and incubated for 72 hours at 37°C, 5% CO2 and 95% humidity followed by an immunofluorescence readout of PNPLA2 (Sigma Aldrich, #HPA055173-25UL). PNPLA2, NTC and PLK1 siRNA were purchased from Dharmacon ((ON-Targetplus SMARTpool Human PNPLA2 siRNA, #L-009003-01-005), (ON-Targetplus SMARTpool Human NTC, #D-001810-10-05), (sGenome PLK1, #M-003290-01)). Immunofluorescence staining for PNPLA2 was performed to confirm knockdown, and results were validated by comparing PNPLA2 expression with and without siRNA treatment.

#### Immunofluorescence staining

Cells were fixed with 4% paraformaldehyde for 15 minutes at room temperature. Cells were blocked with 0.2 M glycine, 0.1 mg/mL saponin, and 30 mg/mL bovine serum albumin (BSA) (all Sigma Aldrich) in PBS for 1 hour. Cells were washed 1x with washing buffer (0,1 mg/ml saponin + 1 mg/ml Bovine serum albumin) using a Blue Washer (Blue Cat Bio). Mouse anti-human ADPN (PNPLA3, C-8, #sc-390252, Santa Cruz) and rabbit anti-human PNPLA2 (Sigma Aldrich, #HPA055173-25UL) primary antibodies and appropriate secondary antibodies conjugated with Alexa-Fluor 647 (Jackson ImmunoResearch) were used. All antibodies were diluted in 0.1 mg/ml Saponin + 1 mg/ml Bovine serum albumin. Incubation with primary antibodies was over night at 4°C. Cells were stained simultaneously with the secondary antibody and Hoechst 33342 (final 2 μg/ml, Biotium) for 1h at room temperature in the dark. For Staining of lipid droplets 38 nM BODIPY was used for 30 minutes at room temperature.

Images were acquired by an Opera Phenix confocal spinning disc microscope system (Revvity, Waltham, MA, USA) with a 20x or 40x water objective at 405 nm (Hoechst), 488 nm (BODIPY) and 647 nm. Quantification was carried out with the Harmony (Revvity, Waltham, MA, USA) or MetaXpress software (Molecular Devices). Normalization, quality control, and fitting curves for EC50 determination of tested compounds were performed with Genedata Screener® for high-content screening and Genedata Condoseo modules (Genedata AG, Basel, Switzerland).

#### Live cell imaging for kinetic studies

Huh7 cells expressing PNPLA3 I148M-tagRFP were seeded at 4000 cells/well in 30 μL media in 384-well imaging plates (Revvity, PhenoPlate) and stored in a 37°C incubator with 5% CO2 and 95% humidity overnight. Cells were then incubated with Bodipy diluted in growth medium (38nM final concentration) for 1h at 37°C with 5% CO2 and 95% humidity. Cells were treated with DMSO control, 1 μM or 5 μM NUV-244, with or without 1 μM Bortezomib or 5 μM MLN7243. Cells were imaged every 15 minutes over a 20-hour period using an Opera Phenix confocal spinning disc microscope system (Revvity) with a 20x water objective at 561 nm (tagRFP) and 488 nm (BODIPY). Images were analyzed using Harmony software. PNPLA3 I148M-tagRFP intensity on lipid droplets was quantified and compared between treatments.

For BFAR siRNA knockdown, cells were seeded at 1000 c/60 μl per well in a 384 well plate (Revvity, Waltham, MA, USA, PhenoPlate 384-well microplate, #6057308) and stored in a 37°C incubator with 5% CO2 and 95% humidity overnight. Then cells were incubated with 10 nM siRNA and Lipofectamine RNAiMAX (Thermo Fisher, Waltham, MA, USA, 1:1000) or control (lipid only, non-targeting-control siRNA) for 48 hours at 37°C, 5% CO2 and 95% humidity followed by BODIPY Staining. BFAR and NTC siRNA were purchased from Dharmacon ((ON-Targetplus SMARTpool Human BFAR siRNA, # L-004386-00-0005), (ON-Targetplus SMARTpool Human NTC, #D-001810-10-05)). After 48h cells were treated with Bodipy diluted in growth medium (38nM final concentration) and incubated for 1h at 37°C with 5% CO2 and 95% humidity. Then DMSO as control (1% final concentration) or compound (1μM or 5μM final concentration) were added to cells. Life cell images were acquired every 15 minutes over 20h by an Opera Phenix confocal spinning disc microscope system (Revvity, Waltham, MA, USA) with a 20x water objective at 561 nm (tagRFP) and 488 nm (BODIPY).

#### Nano differential scanning fluorimetry (nDSF)

Full-length wild-type PNPLA3 (Q9NST1), mutant PNPLA3 (I148M), and PNPLA2 (Q96AD5) were cloned into the pVL1393 vector with an N-terminal Flag-Tag. Proteins were expressed in SF9 cells infected at a multiplicity of infection (MOI) of 1 for 72 hours. Protein purification was performed using a two-step process. The first step involved affinity purification using M2 anti-Flag agarose beads. This was followed by gel filtration chromatography using a Superdex 200 column. Thermal melting experiments were carried out using a Prometheus NT.48 instrument (NanoTemper Technologies). Proteins were prepared in 20 mM Tris-HCl pH 7.4, 100 mM NaCl, 5 % Glycerol, 2 mM DTT and 0.2 mg/mL was used as a final concentration in 50 μL volume. For binding experiments, ligands were added to the mixture at a final concentration of 25 μM, DMSO was used as control. Compounds were tested in triplicates. The temperature gradient was 2°C per min from 20 to 90°C. The intrinsic protein fluorescence at 330 and 350 nm was recorded. Data were analysed using Prometheus NT.48 software. Thermal shifts (ΔTm) were calculated, and Z-scores greater than 6 indicated robust stabilization. Data were processed using Prometheus NT.48 software.

#### Western blotting

For SDS-PAGE and subsequent western blotting cells were harvested in 1x lysis buffer (50 mM Tris-Cl, pH 7.5, 150 mM NaCl, 1 mM EDTA, 1% Triton X-100) supplemented with complete mini protease inhibitor cocktail (Roche). Total protein concentration of samples was determined with a Bicinchoninic Acid (BCA) protein assay. Cell lysates were boiled for 10 min at 99°C with 1x NuPAGE LDS Sample Buffer and loaded onto a 4-12% Tris-Glycine SDS-PAGE gel. For immunoblotting proteins were transferred to a nitrocellulose membrane (0.2 μm pore size, Schleicher & Schuell) and blocked with Intercept (PBS) Blocking buffer (Licor), incubated with primary antibodies anti-PNPLA3 (1:500, C-8 Santa Cruz, SC-390252), anti-PNPLA3 (1:1000, Millipore, MABS2174) and anti-HSP90 (1:1000, Cell Signaling, #4877), and subsequently incubated with secondary antibodies IRDye 680 or IRDye 800 (1:2000; LI-COR Biosciences). Infrared signal was detected using the Odyssey imaging system (Licor).

#### OP9 cells

Transcriptome-wide gene expression profiling was performed using the DRUG-Seq protocol.^40^^,^^41^ Briefly, OP-9 cells were seeded in 384-well CellCarrier Ultra plates at a density of 2500 cells per well. After 24h in culture, adipogenic differentiation was induced using diff1 medium containing 0.5μM dexamethasone, 1μg/ml human insulin, 0.5mM IBMX and 2μM rosiglitazone. Cells were lysed using the DRUG-Seq lysis buffer 16h after the induction of differentiation to capture early transcriptomic changes of adipogenesis. Barcoded cDNA preparation, library synthesis using tagmentation and Illumina sequencing were performed as previously described.^40^^,^^41^ We used STARsolo (2.7.9a) to demultiplex the libraries into individual wells. Count matrices were imported in R and barcodes not present in the whitelist were removed. Differential expression analysis was done with edgeR imposing a fold-change cutoff of 1.2 at the 10% false discovery rate level. GO enrichment analysis was done with clusterProfiler trimming redundant GO terms at the 50% semantic similarity cutoff.

OP-9 cells were maintained at 37°C in a 5% CO2 and 95% air incubator. For differentiation, cells were seeded at 2000 cells and 40μl per well of a 384-well imaging plate (Revvity, Waltham, MA, USA, PhenoPlate 384-well microplate, #6057308) in growth medium and incubated overnight (37°C in a 5% CO2 and 95% air incubator). For differentiation, growth medium was replaced by 60μl differentiation medium 1 (DMEM high glucose with GlutaMAX (Gibco 31966-047 with 10% FCS and 1% Penicillin/Streptomycin), containing 0.5μM Dexamethasone (Tocris, #1126), 1μg/ml human insulin (Merck, #I9278), 0.5mM IBMX (Sigma Aldrich, #I5879), 2μM Rosiglitazone (Sigma Aldrich, #R2408)) and incubated for 3 days (37°C in a 5% CO2 and 95% air incubator). Differentiation medium 1 was then replaced by Differentiation medium 2 (DMEM high glucose with GlutaMAX (Gibco 31966-047 with 10% FCS and 1% Penicillin/Streptomycin), containing 1μg/ml human insulin (Merck, #I9278)) and incubated for 2 days (37°C in a 5% CO2 and 95% air incubator). Cells were then treated with compounds and controls in fresh Differentiation medium 1 and incubated another 24h (37°C in a 5% CO2 and 95% air incubator) and then stained with 2μg/ml Hoechst (Biotium, #40046) and 38nM BODIPY 493/503 (Invitrogen, #D3922) and imaged on an Opera Phenix equipped with a 40x water objective.

#### Cell proliferation assays

Huh7, Huh7 PNPLA3 I148M, Huh7 PNPLA3, Hep3b and LX-2 cells were seeded at a concentration of 1000 cells/well in 60 μl of growth medium on 384-well microtiter plates (Revvity, Waltham, MA, USA, PhenoPlate 384-well microplate, #6057308) and stored in a 37°C incubator with 5% CO2 and 95% humidity overnight. Next day cells were treated with DMSO as control (1% final concentration) or compound (1μM or 5μM final concentration) and cell density was recorded in the Incucyte® life cell imager (Sartorius, Göttingen, Germany) every 4 hours over a period of 5 days. Growth curves were generated by monitoring confluence over time. Proliferative capacity of PNPLA3 I148M-expressing cells was compared to control cells, and the restoration of proliferative fitness was analyzed.

### Quantification and statistical analysis

All statistical analyses for dose-response data were performed using Genedata Screener® or Graphpad Prism. Data were normalized to control values, and the best-fit curves were determined based on standard algorithms for nonlinear regression. For comparisons between experimental conditions, statistical significance was determined using two-way ANOVA or Student’s t-test, where appropriate. All experiments were performed in duplicates or higher. The exact number of replicates (n) for each experiment is noted in the respective figure legends. All data are expressed as mean ± standard deviation (SD), unless otherwise noted in the figure legends.
