# Supplementary material for: Identification of NUV-244 as a PNPLA3 I148M degrading small molecule
Source: iScience. 2025 Apr 8;28(5):112384. doi: 10.1016/j.isci.2025.112384 (PMC12049818; doi:10.1016/j.isci.2025.112384)
Supplement: Document S1. Figures S1–S9 and Table S1 [file mmc1.pdf]

iScience, Volume *28*

Supplemental information

Identification of NUV-244 as a PNPLA3 I148M degrading small molecule

Patrick Steigemann, Nico Braeuer, Vera Puetter, Nina Zablowsky, Katrin Juenemann, Franz von Nussbaum, Ralf Lesche, Nicole Dittmar, David Schaller, Zuzanna Makowska, Filippos Klironomos, Susanne Schwarz, Daniela Launhardt, Benjamin Bader, Martin Lange, Holger Steuber, Mary Helen Black, Jonathan S. Packer, Stefano Romeo, Stephan Fasler, Lisa Bedford, and Frederick E. Dewey

a. **b.**

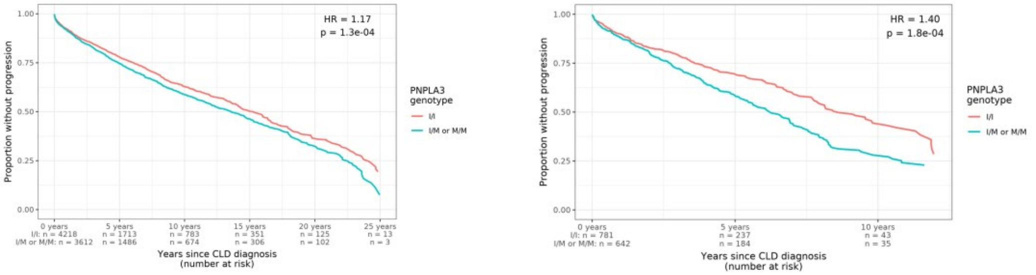

Supplemental Fig. 1: Association of PNPLA3 148M with progression from initial chronic fatty liver disease diagnosis to cirrhosis

a. Kaplan-Meier analysis of time to progression from a subject's first inpatient chronic fatty liver disease (CLD) diagnosis to a first inpatient cirrhosis diagnosis in the UK Biobank.
b. Kaplan-Meier analysis of time to progression from a subject's first inpatient chronic fatty liver disease (CLD) diagnosis to a first inpatient cirrhosis diagnosis in the UK Biobank among study participants with type 2 diabetes.

Predictor	Hazard ratio [95% confidence interval]	P value
PNPLA3 148M	1.16 [1.09-1.23]	4.0x10-06
TM6SF2 167K	1.12 [1.02-1.23]	1.9x10-02
HSD17B13 splice variant	0.93 [0.87-0.99]	3.4x10-02
Prior diabetes	1.52 [1.37-1.68]	1.6x10-15
BMI (units of 5 kg/m2)	0.95 [0.91-0.99]	7.0x10-03

Supplemental Table 1:

Hazard ratios for predictors of time to progression from a subject’s first inpatient CLD diagnosis to a composite endpoint comprised of inpatient cirrhosis, hepatocellular carcinoma, liver transplant, or liver-related mortality in the UK Biobank. Separate Cox proportional hazards models were tested for each predictor adjusted for age at CLD diagnosis, sex, genotyping chip, and the first six principal components of ancestry as covariates.

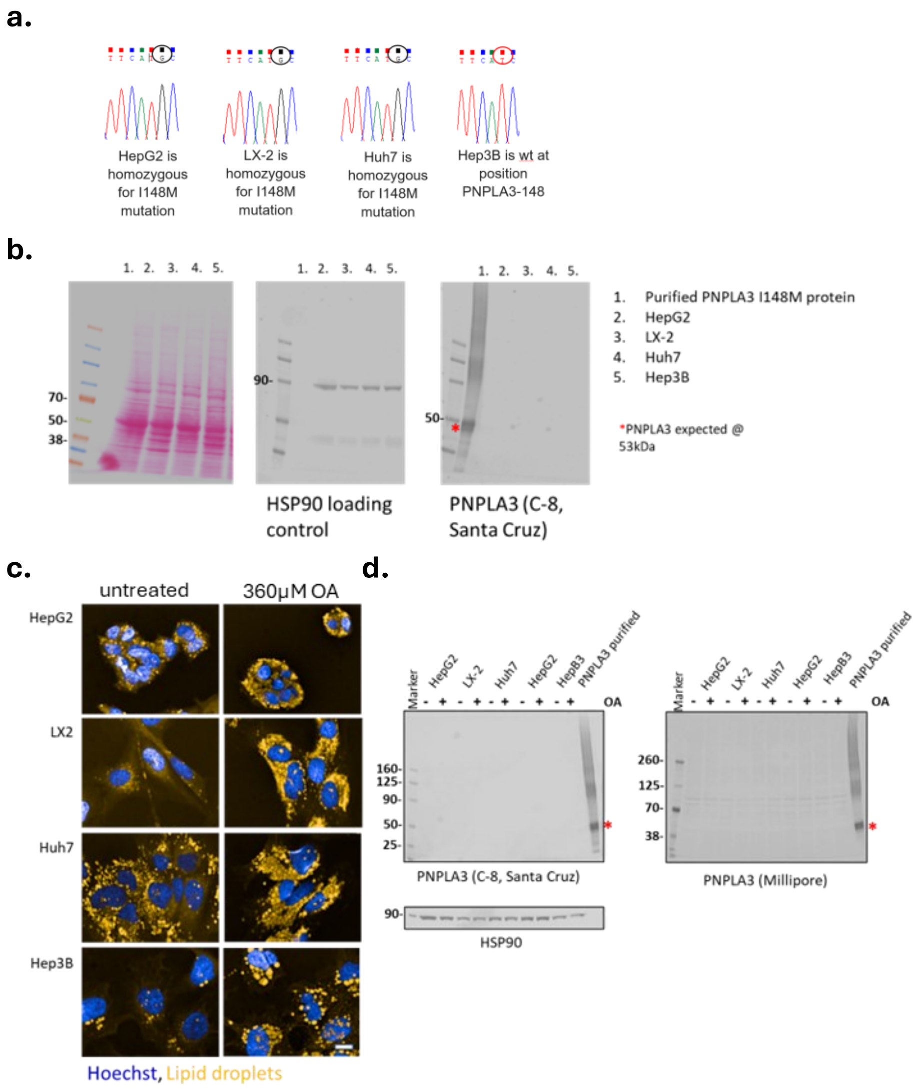

Supplemental Fig. 2: Cell line characterization

a. Sequencing results on the PNPLA3 locus in different cell lines.

b. Purified PNPLA3 I148M can be detected by WB using Santa Cruz C-8 anti-PNPLA3 antibody but no PNPLA3 signal can be detected in the cell lines tested

c. Lipid droplet staining in normal medium and after 24h in medium containing 360µM oleic acid. Scale bar ~10µm.

d. Western Blot using two different antibodies against PNPLA3 show no signal for PNPLA3 in normal medium or fed cells. Purified PNPLA3 protein shows a band at the correct size.

a.

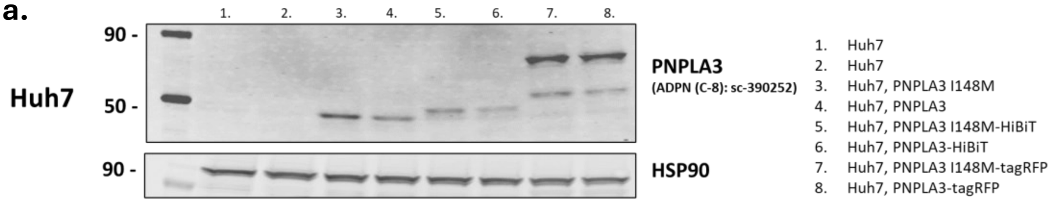

Supplemental Fig. 3: Cell line characterization

a. Western blot using anti-PNPLA3 antibody to detect PNPLA3 expression in Huh7 cells. HSP90 was used as loading control. While no protein could be detected in parental cell lines, recombinant, stably transduced cell lines generated with expression vectors for different PNPLA3 variants showed detectable expression at expected sizes.

a.

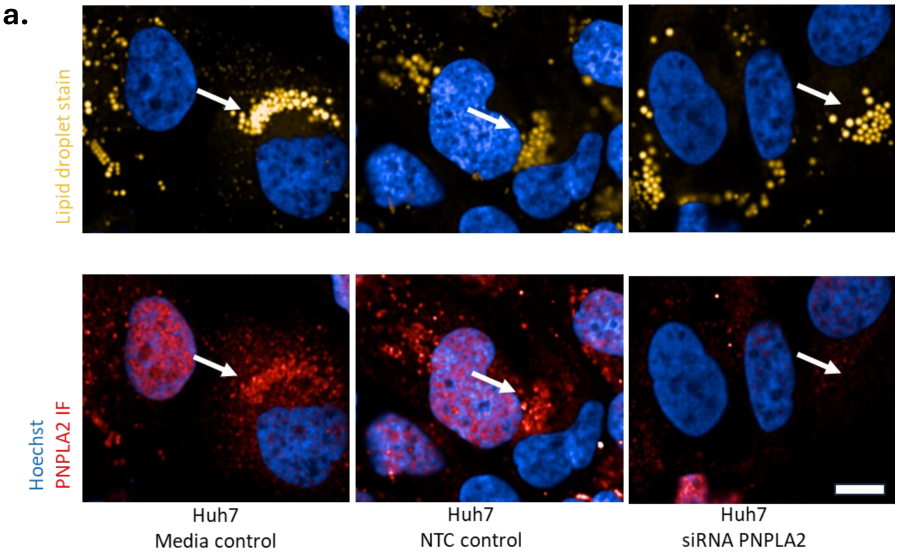

Supplemental Fig. 4: PNPLA2 IF validation

a. Validation of PNPLA2 antibody staining by siRNA. PNPLA2 siRNA (72h) significantly decreases PNPLA2 antibody staining on lipid droplets. Scale bar ~10µM.

a.

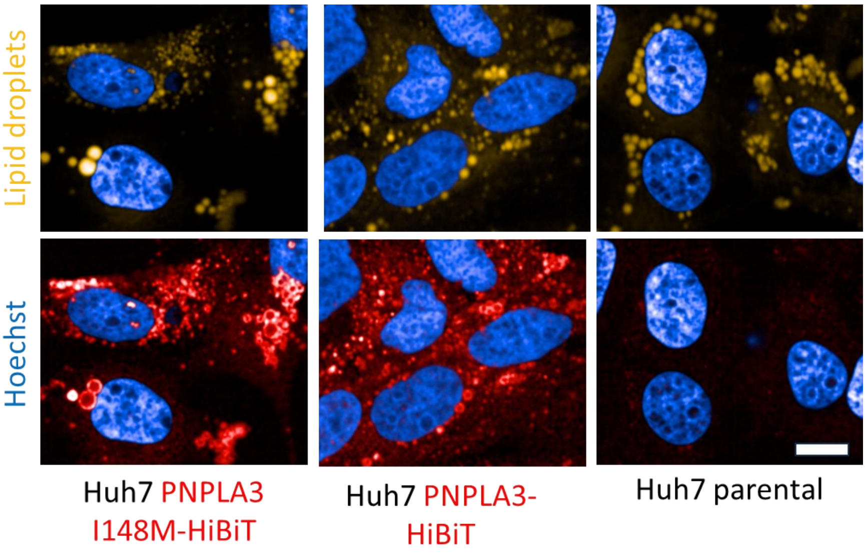

Supplemental Fig. 5: cell line characterization

a. PNPLA3 I148M-HiBiT predominantly localizes to lipid droplets in Huh7 cells. Huh7 cells expressing PNPLA3 I148M-HiBiT were stained by immunofluorescence with an anti-PNPLA3 antibody. No signal is detected on Huh7 parental cells. Scale bar ~10µm.

a.

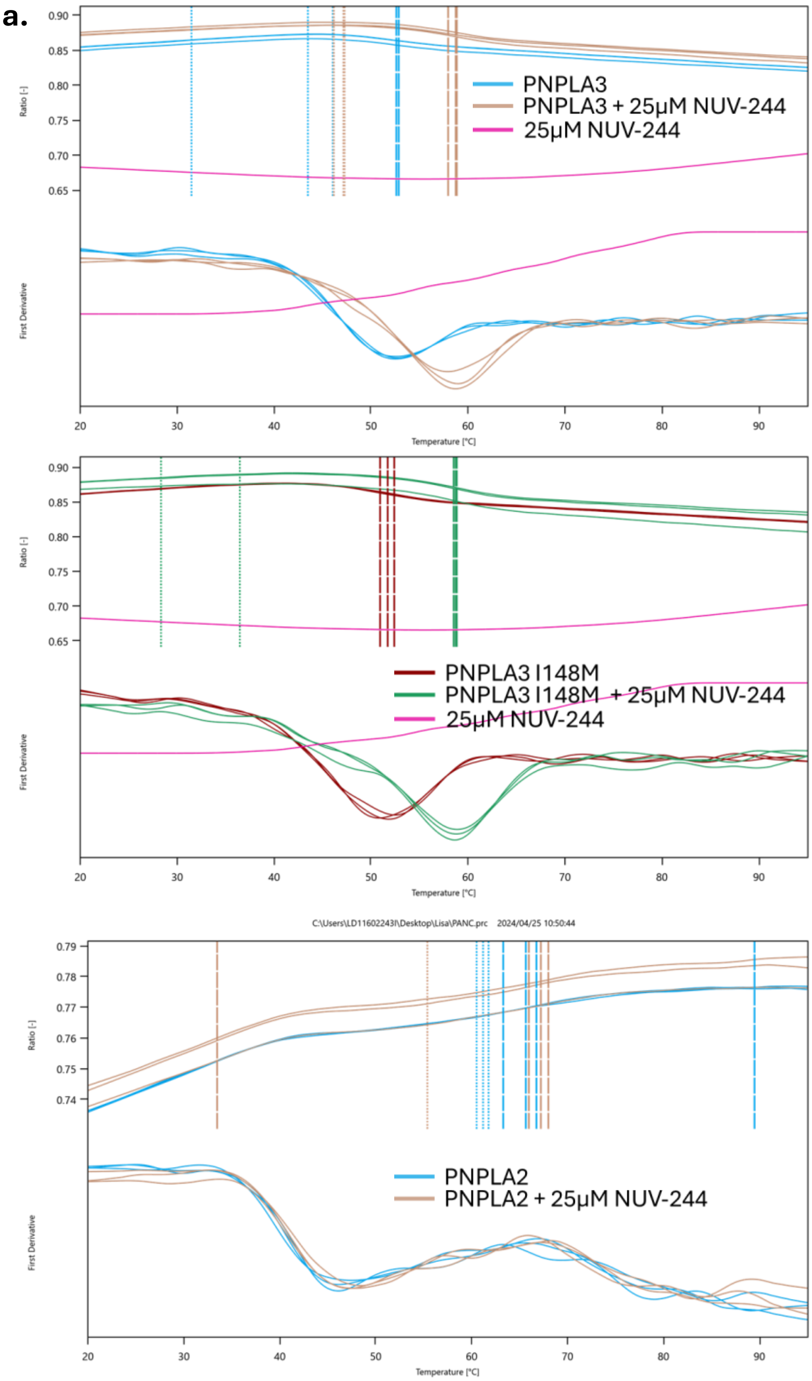

Supplemental Fig. 6: nano Differential Scanning Fluometry (nDSF) analysis

a. nDSF unfolding curves of PNPLA3 I148M, PNPLA3 WT and PNPLA2. Ratio of fluorescence at 330/350 is shown in the upper and the first derivative is plotted in the bottom half of each plot. Inflection points (equivalent to the Tm) are shown as vertical lines (n=3 per condition). Similar data was obtained in >2 independent experiments.

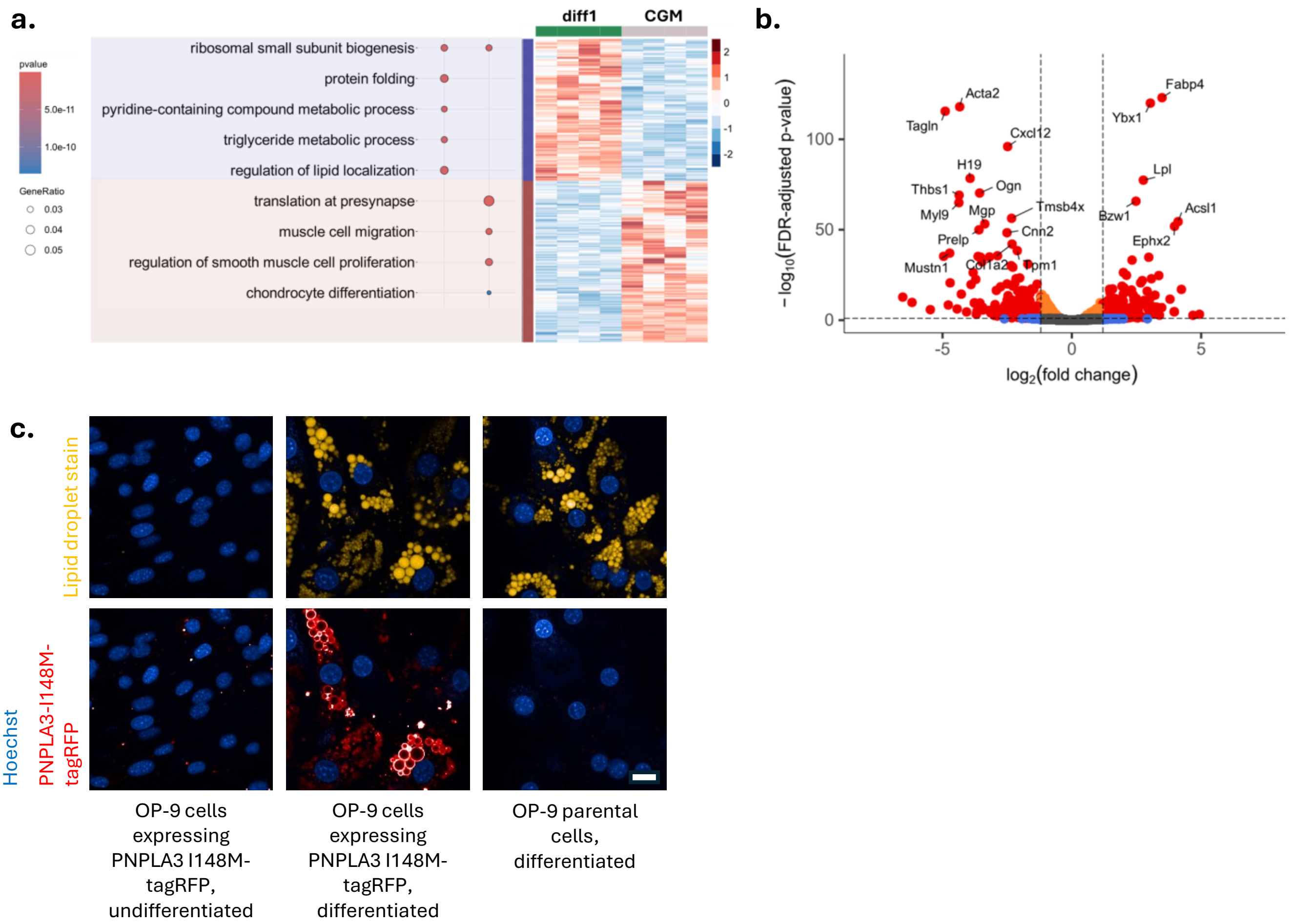

Supplemental Fig. 7: OP-9 differentiation and PNPLA3 I148M-tagRFP localization

a. The heatmap shows the expression of significantly differentially regulated genes between the parental OP9 cells that have been treated for 16h with diff1 medium (IBMX, dexamethasone, insulin, rosiglitazone) and cells left in their complete growth media (CGM), each in biological quadruplicate. Differentially regulated genes formed two clusters, each with a unique signature of Gene Ontology Biological Process terms.
b. The volcano plot shows the log2 fold change and –log10 false discovery rate of all detected genes for the comparison between the parental OP9 cells that have been treated for 16h with diff1 medium (IBMX, dexamethasone, insulin, rosiglitazone) and cells left in their complete growth media (CGM), each in biological quadruplicate. A total of 1409 genes were found significantly differentially expressed between the two conditions (in red). c. Differentiated OP-9 cells accumulate large lipid droplets. PNPLA3 I148M-tagRFP predominantly localizes to the surface of lipid droplets in PNPLA3 I148M-tagRFP expressing cells. Scale bar ~10µm.

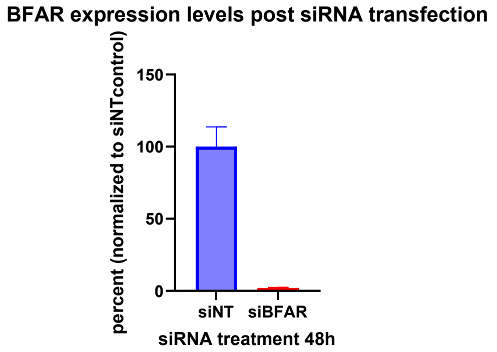

Supplemental Fig. 8: validation of BFAR siRNA knockdown by RT-PCR

Relative BFAR mRNA expression levels were quantified by qPCR following treatment with non-targeting siRNA (siNT) and siRNA targeting BFAR (siBFAR). GAPDH was used as the internal normalization control to calculate ΔCt values (BFAR Ct - GAPDH Ct) for each condition. The average ΔCt for siNT was used as the reference for calculating ΔΔCt values (ΔCt - mean ΔCt of siNT). Relative expression levels were then determined using the 2^-ΔΔCt method and expressed as a percentage of siNT control (set to 100%). BFAR expression was reduced to 2.0% ± 0.4% (mean ± SD, n=3) compared to the siNT control, demonstrating highly effective knockdown. ΔCt values for siNT ranged from 6.40 to 6.89, while for siBFAR they ranged from 11.99 to 12.71.

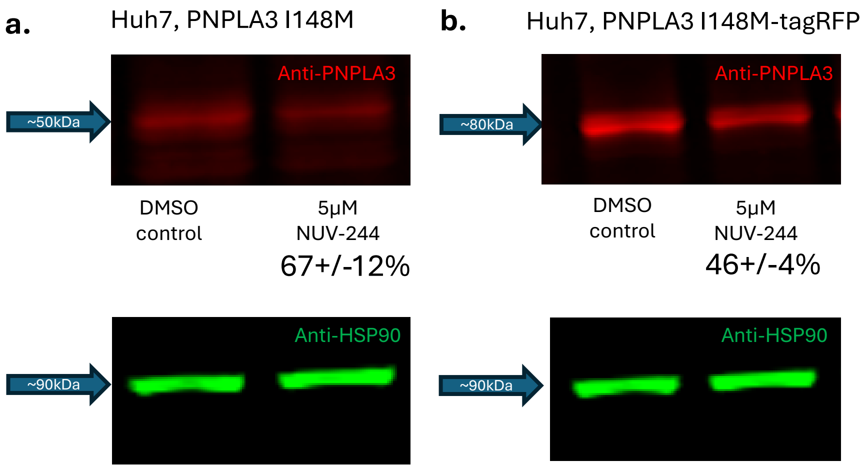

Supplemental Fig. 9: Western Blot NUV-244

a. Huh7 cell expressing PNPLA3 I148M or b. PNPLA3 I148M-tagRFP were treated for 24h with DMSO or 5µM NUV-422. Western blot was performed using an anti-PNPLA3 antibody (SC-390252). PNPLA3 bands at 53kDa (unmodified in a.) or 80 kDa (PNPLA3 I148M-tagRFP) were quantified (signal above local background, normalized to loading control using LICOR Odyssey CLx / Image Studio) on two independent Western Blots and percent of intensity compared to DMSO controls calculated (Mean +/- Stdev). Loading controls: anti-HSP90.
